# HER2-targeting antibody–drug conjugate RC48 alone or in combination with immunotherapy for locally advanced or metastatic urothelial carcinoma: a multicenter, real-world study

**DOI:** 10.1007/s00262-023-03419-1

**Published:** 2023-03-10

**Authors:** Meiting Chen, Kai Yao, Manming Cao, Hao Liu, Cong Xue, Tao Qin, Lingru Meng, Zhousan Zheng, Zike Qin, Fangjian Zhou, Zhuowei Liu, Yanxia Shi, Xin An

**Affiliations:** 1 Department of Medical Oncology, Sun Yat-Sen University Cancer Center, State Key Laboratory of Oncology in South China, Collaborative Innovation Center for Cancer Medicine, No. 651, Dongfeng East Road, Yuexiu District, Guangzhou, 510060 China; 2 Department of Urology, Sun Yat-Sen University Cancer Center, State Key Laboratory of Oncology in South China, Collaborative Innovation Center for Cancer Medicine, No. 651, Dongfeng East Road, Yuexiu District, Guangzhou, 510060 China; 3 Department of Medical Oncology, Zhujiang Hospital, Southern Medical University, Guangzhou, 510282 China; 4 Department of Urology, Sun Yat-Sen Memorial Hospital, Sun Yat-Sen University, Yuexiu District, 107 Yanjiangxi Road, Guangzhou, 510120 Guangdong China; 5 Department of Medical Oncology, Sun Yat-Sen Memorial Hospital, Sun Yat-Sen University, 107 Yanjiangxi Road, Yuexiu District, Guangzhou, 510120 Guangdong China; 6 Ward 2, Department of Oncology, Hainan Cancer Hospital, Haikou, China; 7 Department of Medical Oncology, the First Affiliated Hospital, Sun Yat-Sen University, Guangzhou, 510060 China

**Keywords:** Metastatic urothelial carcinoma, Anti-HER2 antibody–drug conjugate, RC48, Real-world study

## Abstract

**Background:**

Phase II trials showed the efficacy of anti-HER2 RC48-ADC (disitamab vedotin) for HER2-positive metastatic urothelial carcinoma (UC). This study evaluated RC48 alone verses in combination with immunotherapy for locally advanced or metastatic UC using real-world data.

**Methods:**

This retrospective, multicenter, real-world study included patients with locally advanced or metastatic UC who received RC48 in five hospitals in China between July 2021 and April 2022. The outcomes were progression-free survival (PFS), overall survival (OS), objective response rate (ORR), disease control rate (DCR), and adverse events.

**Results:**

Thirty-six patients were included. The patients were 47–87 years, and 26 (72.2%) were male. Eighteen patients received RC48 alone, and 18 received RC48 combined with a programmed death-1 antibody. The median PFS was 5.4 months. The median OS was not reached. The 6-month and 1-year PFS rates were 38.8% and 15.5%, respectively. The 1-year OS rate was 79.6%. Fourteen (38.9%) patients achieved a partial response, and the ORR was 38.9%. Eleven patients had stable disease, and the DCR was 69.4%. The median PFS for patients who received RC48 combined with immunotherapy and those who received RC48 alone was 8.5 and 5.4 months, respectively. The main treatment-related adverse events included anemia, hypoesthesia, fatigue, and elevated transaminase. No treatment-related death occurred.

**Conclusion:**

RC48 alone or combined with immunotherapy might benefit patients with locally advanced or metastatic UC, regardless of impaired renal function.

**Supplementary Information:**

The online version contains supplementary material available at 10.1007/s00262-023-03419-1.

## Introduction

Locally advanced or metastatic urothelial carcinoma (UC) has a poor prognosis despite discovering novel therapies [1]. Cisplatin-containing therapy remains the first-line therapy for metastatic UC, with an overall response rate (ORR) of 44.6% and median progression-free survival (PFS) and overall survival (OS) of 7.8 and 14.0 months, respectively [2]. Still, approximately 50% of the patients with metastatic UC are ineligible to receive cisplatin-based chemotherapy due to impaired renal function, poor performance status, or comorbidities. The alternative therapies include carboplatin-based chemotherapy and immunotherapy, but the outcomes are unsatisfactory, with a lower ORR (at 28–36%) and shorter PFS for patients with metastatic UC [2–4].

Immune checkpoint inhibitors (ICIs) are the standard second-line treatment for patients with UC [2, 5], but only approximately 25% of the patients achieve a response to ICIs [6, 7]. The treatment of patients who progressed after platinum-based chemotherapy and ICIs mainly includes enfortumab vedotin (EV) and sacituzumab govitecan (SG) [8, 9], but neither EV nor SG is available in China. Therefore, effective treatment options are necessary for metastatic UC.

HER2 overexpression is commonly observed in UC and is associated with poor survival [10, 11]. Recently, anti-HER2 antibody–drug conjugates (ADCs), including RC48 (disitamab vedotin) and trastuzumab deruxtecan, showed certain efficacy in metastatic UC patients after platinum-based chemotherapy and ICIs [9, 12, 13]. The phase II RC48-C005 trial reported the excellent antitumor activity of RC48-C005 monotherapy in patients with metastatic UC after failure to at least one line of systemic treatment, with an ORR of 51.2% and median PFS of 6.9 months [13]. In the RC48-C014 trial, 32 patients with metastatic UC treated with RC48 combined with toripalimab, an anti-programmed cell death-1 (PD-1) antibody, ORR was 75% for overall population, and 80% for first-line previously untreated patients [14]. Therefore, RC48, available in China, might be an appropriate treatment option for metastatic UC, either as first- or further-line therapy.

Although randomized controlled trials (RCTs) can prove the efficacy of a drug within strictly controlled experimental conditions, RCTs often suffer from selection biases (e.g., exclusion of older patients and those with comorbidities) that limit the applicability of their results. On the other hand, real-world studies provide important information on a drug’s efficacy and safety in the actual population of patients. The two types of studies are considered complementary [15, 16]. Unfortunately, real-world data on the role of RC48 in metastatic UC are lacking. Therefore, this study explored the RC48 alone or in combination with immunotherapy for locally advanced or metastatic UC with real-world data.

## Materials and methods

### Study design and patients

This retrospective, multicenter, real-world study included patients with locally advanced or metastatic UC who received RC48 at the Sun Yat-Sen University Cancer Centre (SYSUCC), the First Affiliated Hospital of Sun Yat-Sen University, Sun Yat-Sen Memorial Hospital, Zhujiang Hospital of the Southern Medical University, and Hainan General Hospital between July 2021 and April 2022. The inclusion criteria were (1) histologically confirmed UC, (2) received RC48, (3) available response assessments, and (4) adequate cardiac, bone marrow, and hepatic functions apart from organ function affected by their disease. The study protocol was approved by the ethical committee of the Sun Yat-Sen University Cancer Centre (No. B2022-271-01). The requirement for individual informed consent was waived by the committee because of the retrospective nature of the study.

### Data collection and definitions

The data were extracted from the medical charts and included the patients’ demographics, tumor characteristics, treatment, standard laboratory tests, and image scans. Generally, the patients were treated with RC48 until disease progression, intolerable toxicity, or death. Dose modification was made in case of treatment-related adverse events (TRAEs) until these events resolved to grade 0/1 or to baseline. The use of a PD-1 antibody was at the physicians’ discretion and was given according to their experience. HER2 expression was mainly detected by immunohistochemistry (IHC). The IHC scores were assessed according to the HER2 test guidelines for breast cancer [17]. The HER2 gene amplification could also be evaluated by fluorescence in situ hybridization (FISH), which was compliant with the HER2 test guidelines for breast cancer [17]. Some patients were tested for HER2 gene mutation by next-generation sequencing (NGS).

The adverse events (AEs) were graded according to the Common Terminology Criteria for Adverse Events version 5.0. The relation of each AE with RC48 and treatments was considered possibly, probably, or likely related to treatment and estimated as the proportion of all toxicity-evaluable cycles in which toxicity was observed.

The objective response was a response sustained for a minimum of two consecutive imaging evaluations at least 4 weeks apart. The disease was evaluated using RECIST version 1.1 for response assessment. CT was routinely performed at baseline and every 6 weeks. Follow-up CT scans data were collected for 2 years or until progressive disease (PD).

Survival was measured from initiation of therapy until the event of interest (death or progression). The disease control rate (DCR), ORR, PFS, OS, and AEs were analyzed. The DCR was calculated as the proportion of patients achieving a complete response (CR), partial response (PR), or stable disease (SD). The ORR was calculated as the proportion of patients achieving a CR or a PR. The duration of response (DOR) is defined as the time from the first evaluation of CR, PR, or SD to PD. Creatinine clearance (CrCl) was obtained from the routine biochemistry examinations. Follow-up was censored on August 10, 2022.

### Statistical analysis

SPSS 25.0 (IBM, Armonk, NY, USA), Prism 5.01 (GraphPad Software Inc., San Diego, CA, USA), and R 4.0.2 (The R Project for Statistical Computing, www.r-project.org) were used for statistical analysis. The study population for all analyses included the patients included in the study who had received at least one dose of RC48. Descriptive statistics were used to summarize patient characteristics, treatment administration, antitumor activity, and safety. OS and PFS were analyzed using the Kaplan–Meier method and Cox proportional hazard models. Two-sided *P* < 0.05 were considered statistically significant.

## Results

Thirty-six eligible patients were included. The patients were aged from 47 to 87 years, with 14 (38.9%) being > 65 years, and 26 (72.2%) of them were male. Among the 27 (75%) patients who underwent primary surgery, eight (22.2%) relapsed within 1 year after neoadjuvant or adjuvant chemotherapy. HER2 expression was positive (IHC 3 +, or 2 +) in 28 (77.8%) patients. In 17 (47.2%) patients with available PD-L1 detection results, the rate of PD-L1 positivity (> 1%) was 52.9%. Among the 36 patients, eight (22.2%) underwent NGS (Table 1).

**Table 1:** Characteristics of the patients

Characteristics	Values
Male sex, *n* (%)	26 (72.2)
Age (years)	
Median (range)	62.4 (47–87)
HER2 expression, *n* (%)	
IHC 1 +	7 (19.4)
IHC 2 +	23 (63.9)
IHC 3 +	5 (13.9)
IHC 0^a^	1 (2.8)
PD-L1 expression, *n* (%)	
≥1%	9 (52.9)
< 1%	8 (47.1)
NA	19
Prior therapy, *n* (%)	
Median (range)	2.58 (0–6)
RC48 as first-line therapy	9 (25.0)
RC48 as second-line therapy	10 (27.8)
RC48 after second-line therapy	17 (47.2)
Prior PD-1 immunotherapy	22 (61.1)
Prior platinum-based chemotherapy	23 (63.9)
Relapsed within 12 months of neoadjuvant or adjuvant platinum-based chemotherapy	8 (22.2)
Prior enfortumab vedotin	2 (5.6)
Baseline creatinine clearance	
≥ 60 ml/min	20 (55.5)
30–60 ml/min	11 (30.6)
< 30 ml/min	5 (13.9)
ECOG PS	
0	4 (11.1)
1	26 (72.2)
2	6 (16.7)
Pathologic variants	
Urothelial carcinoma/transitional cell	30 (83.3)
Urothelial carcinoma with squamous differentiation	5 (13.9)
Urothelial carcinoma with glandular differentiation	1 (2.8)
Prior locoregional curative treatments, *n* (%)	
Surgery	27 (75.0)
Radiotherapy	2 (5.6)
Prior neoadjuvant/adjuvant chemotherapy, *n* (%)^b^	
Yes	9 (33.3)
No	18 (66.7)
Primary lesion, *n* (%)	
Renal pelvis	8 (22.2)
Ureter	11 (30.6)
Bladder	17 (52.8)
Metastasis site, *n* (%)	
Local relapse	18 (51.4)
Brain	1 (2.8)
Lymph node metastasis	27 (75.0)
Lung	12 (33.3)
Bone	13 (36.1)
Liver	15 (41.7)
Adrenal gland	3 (8.3)
Peritoneal or omental implantation	7 (19.4)

*IHC* immunohistochemistry, *PD-1* programmed cell death 1, *PD-L1* programmed cell death ligand, *ECOG* Eastern Cooperative Oncology Group, *PS* performance status

^a^This patient had HER2 insertion mutation (Py772_A775dup) at exon 20

^b^Only for patients who had been underwent curative surgery

Eighteen patients received RC48 alone, and 18 received RC48 combined with a PD-1 antibody, including toripalimab, tislelizumab, pembrolizumab, envafolimab, and sintilimab. Nine patients received RC48 as first-line therapy due to ineligible for cisplatin-based chemotherapy (5 patients), ineligible for both cisplatin and carboplatin (3 patients), and refusing to receive chemotherapy (1 patient) (Supplementary Table S1). At the end of the follow-up, 13 (36.1%) patients were still on treatment. All 36 patients had at least one response evaluation, 14 (38.9%) achieved a PR, and 11 patients had an SD. The ORR was 38.9%, and the DCR was 69.4%. The ORRs of the patients treated with RC48 alone and RC48 combined with PD-1 were both 38.8%. The ORR was 60.0% (3/5), 39.1% (9/23), and 28.6% for patients with HER2 3 +, 2 +, 1 + expression, respectively. The ORR was 44.4% (4/9) in patients positive for PD-L1 and 37.5% (3/8) in PD-L1-negative patients. For the nine patients received RC48 as first-line therapy, the ORR was 55.6% (5/9), compared with 33.3% (9/27) in patients received RC48 as second- and later-line therapy.

**Fig. 1:**
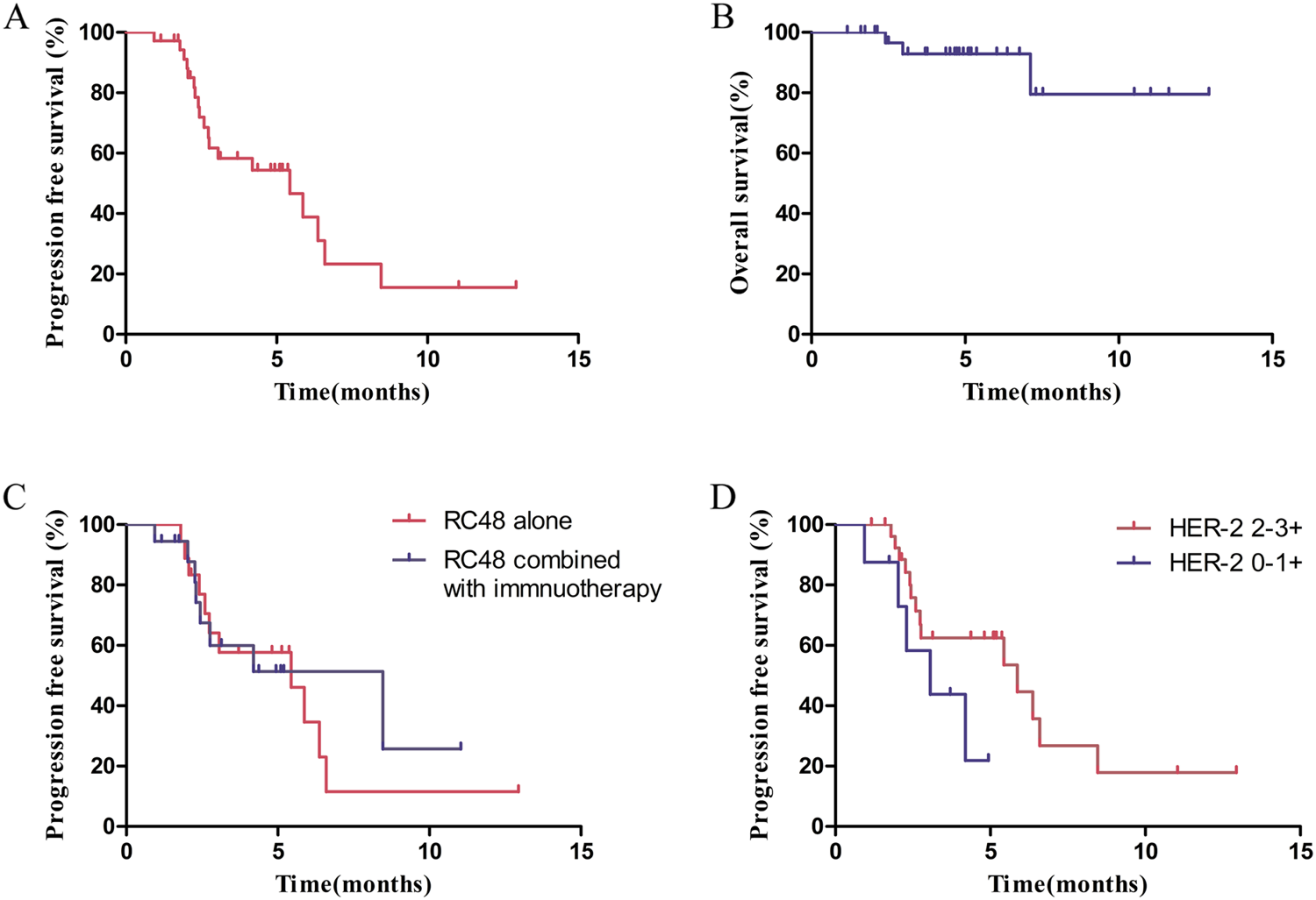
Progression-free survival (PFS) (**A**) and overall survival (OS) (**B**) of the patients receiving RC48. PFS of the patients received RC48 alone and combined with immunotherapy (**C**). PFS of the patients with HER2 1 + compared with HER2 2 + and 3 + (**D**)

**Fig. 2:**
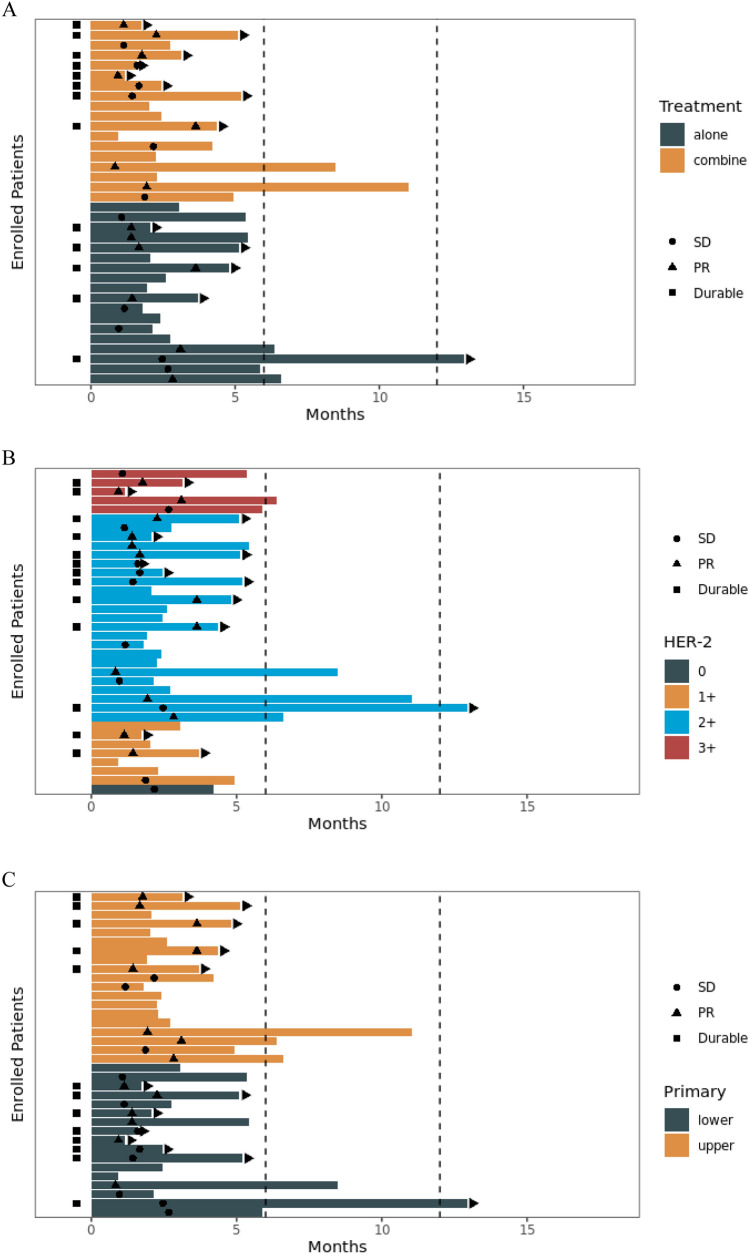
Swimmer plot of the patients receiving RC48 alone and combined with immunotherapy (**A**). Swimmer plot of the patients with different HER2 expressions (**B**). Swimmer plot of the patients with primary site located in lower and upper urinary tract (**C**)

**Fig. 3:**
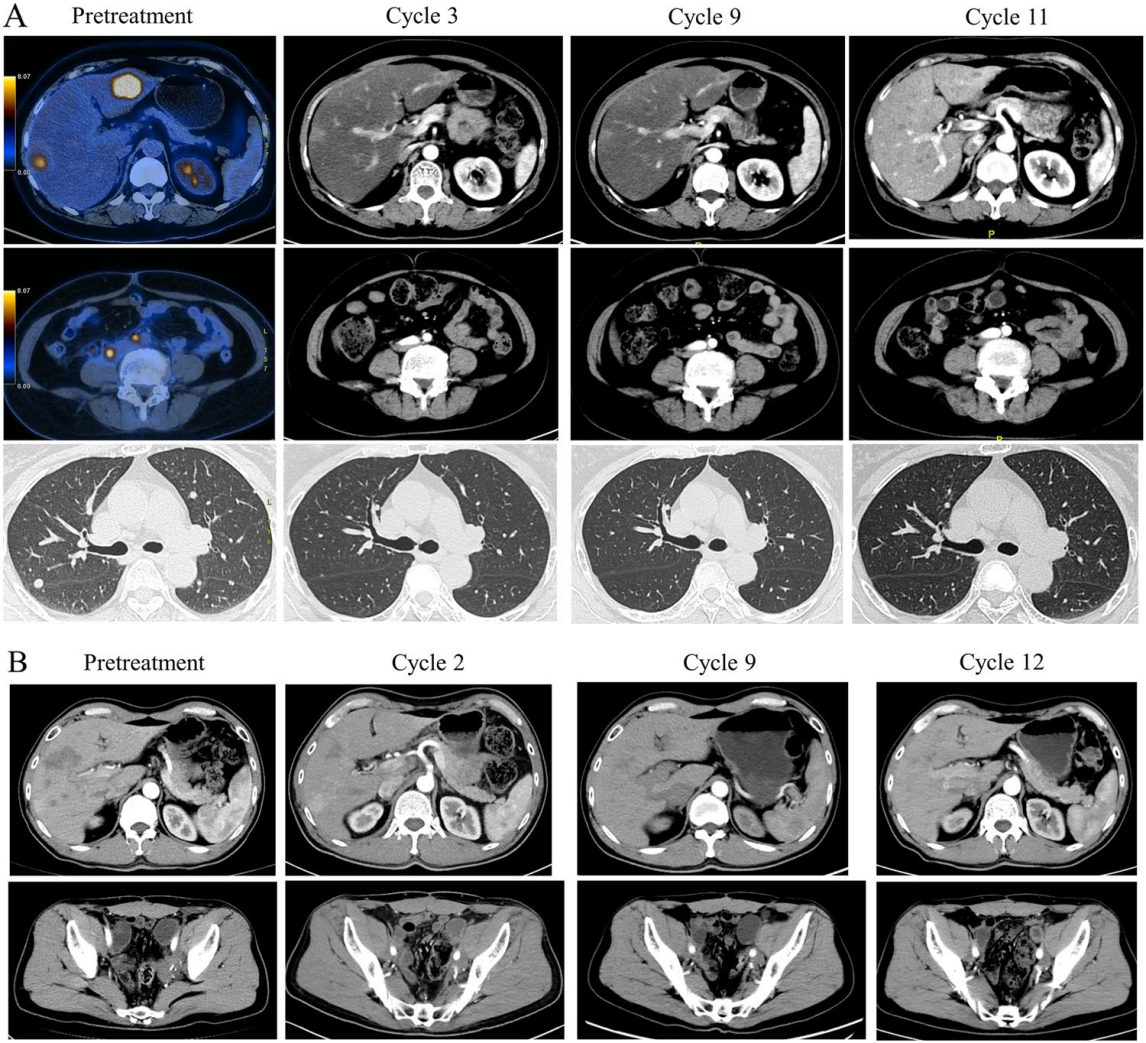
Representative pre-treatment and post-treatment images in patients #6 (**A**) and #10 (**B**)

The median PFS was 5.4 months, and the median OS was not reached. The 6-month and 1-year PFS rates were 38.8% and 15.5%, respectively (Fig. 1A). The 1-year OS rate was 79.6% (Fig. 1B). The median PFS for patients who received RC48 combined with immunotherapy and those treated with RC48 alone was 8.5 and 5.4 months, respectively (HR = 1.15, 95% CI 0.46–2.88, *P* = 0.75) (Fig. 1C). The median PFS for patients with HER2 2 +/3 + expression and those with HER2 1 +/0 expression was 5.9 and 3.0 months, respectively (*P* = 0.11, HR = 0.34, 95% CI 0.09–1.28) (Fig. 1D). The PFS in patients received RC48 as first line was 6.6 months, compared with 4.2 months in those with second- and later-line treatment (*P* = 0.09) (Figure S1). The swimmer plot for all patients is shown in Fig. 2. For the 25 patients who achieved SD or PR, the DOR was 4.0 months. Patient #6 showed the best tumor response of PR (Fig. 3A) and a DOR of 9.1 months, but she discontinued RC48 due to grade 2 hypoesthesia. Patient #10 relapsed 11 months after adjuvant cisplatin and gemcitabine and rapidly progressed after combination of EV and pembrolizumab. Then, he was treated with RC 48 monotherapy and achieved good PR with a DOR of 7.6 months (Fig. 3B).

NGS in eight patients showed that an FGFR amplification or mutation was detected in four patients. Two patients had a TP53 mutation. One patient had a high tumor mutation burden (TMB), while the others presented low TMB. HER-2 amplification was detected in one patient (#26) who had HER2 2 + by IHC. No HER-2 gene amplification was detected in the rest of them. A HER2 insertion mutation (Py772_A775dup) at exon 20 was detected in patient #14, who was HER2 0 + by IHC. This patient received RC48 and immunotherapy and achieved SD with significant symptom improvement (Table 2).

**Table 2:** Summary of NGS results of eight patients

Patient #	TMB	MSI	Major NGS results	Treatment	PFS (months)
3	Low	MSS	FGFR3 pY373C mutation	RC48	12.9
10	Low	MSS	FGFR3 pS249C mutation	RC48 and pembrolizumab	8.47
11	High	MSS	CDKN2A pD84N mutation, RB1 pK327 mutation, PTEN pP339K mutation	RC48 and tislelizumab	2.27
12	Low	MSS	TP53 pC176Y mutation, KEAP1 pR320Q mutation, MLL2 mutation, CSMD2 mutation, FGFR2 mutation, EPHA5 mutation, MAP2K1 mutation, FLT3 amplification, PLT1 amplification, NBN amplification	RC48	2.40
13	Low	MSS	AKT1 pE17K mutation, RAF1 amplification	RC48	1.80
14	Low	MSS	HER2 insertion mutation (Py772_A775dup) at exon 20, CDKN1A pD86fs mutation	RC48 and sintilimab	4.20
26	NA	NA	ERBB2 amplification and TP53 pR175H mutation	RC48 and toripalimab	1.17
31	NA	NA	FGFR amplification	RC48	2.07

*TMB* tumor mutation burden, *MSI* microsatellite instability, *NGS* next-generation sequencing, *PFS* progression-free survival, *MSS* microsatellite stable

The AEs in patients treated with RC48 alone and combined with immunotherapy are listed in Table 3. Overall, grade 3–4 AEs occurred in five (27.8%) patients received the combination treatment (including one grade 3 anemia, one grade 3 hypoesthesia, one grade 3 hypoalbuminemia, one grade 3 urinary tract infection, and one autoimmune encephalitis), compared with two (11.1%) patients treated with RC 48 alone (two grade 3 anemia). Neither grade 4 nor treatment-related death occurred in the all patients. The CrCl was < 50 mL/min in eight patients and < 30 mL/min in five patients before receiving RC48. The dosage and frequency remained the same as a regular treatment; renal function was closely monitored before each cycle of treatment. Patient #1 showed increase of serum creatinine. She had a history of renal transplantation 2 years ago and still in treatment of immunosuppressed drugs. Her baseline CrCl was 29 mL/min, and grade 1 increase of serum creatinine was observed during the first two cycle of RC48 monotherapy, then serum creatinine gradually slowed down, and CrCl remained approximate 30 mL/min during the subsequent treatment. This lady also showed a PR response. Patient #4 was diagnosed with autoimmune encephalitis and urinary tract infection. He was consulted for confusion and fever after the second cycle of RC48 and tislelizumab. Magnetic resonance imaging showed no brain metastasis, and the tumor evaluation was SD after two cycles. RC48 was discontinued; then, he suffered from a femoral neck fracture and septic shock and was treated with the best supportive care.

**Table 3:** Summary of the adverse events

Events, *n* (%)	Patients (*n* = 36)		Patients treated with RC48 alone (*n* = 18)		Patients treated with RC48 combined with immunotherapy (*n* = 18)	
	Any grade	Grade 3–4	Any grade	Grade 3–4	Any grade	Grade 3–4
Any adverse event	36 (100)	6 (16.7)	18 (100)	2 (11.1)	18 (100)	5 (27.8)
Anemia	20 (55.6)	3 (8.3)	8 (44.4)	2 (11.1)	12 (66.7)	1 (5.6)
Leukopenia	5 (13.9)	0	1 (5.6)	0	4 (22.2)	0
Thrombocytopenia	1 (2.8)	0	1 (5.6)	0	0	0
Fatigue	12 (33.3)	0	7 (38.9)	0	5 (27.8)	0
Hypoesthesia	12 (33.3)	1 (2.8)	8 (44.4)	0	4 (22.2)	1 (5.6)
Dyspepsia	9 (25.0)	0	6 (33.3)	0	3 (16.7)	0
Nausea	4 (11.1)	0	2 (11.1)	0	2 (11.1)	0
Diarrhea	2 (5.6)	0	0	0	2 (11.1)	0
Constipation	6 (16.7)	0	3 (16.7)	0	3 (16.7)	0
Serum creatinine increased	2 (5.6)	0	1 (5.6)	0	1 (5.6)	0
Elevated transaminases	8 (22.2)	0	3 (16.7)	0	5 (27.8)	0
Hypoalbuminemia	15 (41.7)	1 (2.8)	8 (44.4)	0	7 (38.9)	1 (5.6)
Hyponatremia	6 (16.7)	0	3 (16.7)	0	3 (16.7)	0
Urinary tract infection	3 (8.3)	1 (2.8)	0	0	3 (16.7)	1 (5.6)
Pruritus	2 (5.6)	0	1 (5.6)	0	1 (5.6)	0
*Immune-related AEs*						
Autoimmune encephalitis	1 (5.6)	1 (5.6)	–	–	1 (5.6)	1 (5.6)
Myositis	1 (5.6)	0	–	–	1 (5.6)	0

## Discussion

The results showed that RC48 alone or combined with immunotherapy showed excellent antitumor activity in patients with locally advanced or metastatic UC. The results suggest that RC48 might be safe even in patients with moderate or severe renal impairment. The results could help delineate the clinical benefits of RC48 in patients with locally advanced or metastatic UC and help define its indications.

Besides breast cancer, HER2 is a potential target in many solid tumors, including metastatic UC [18], gastric cancer [19], and non-small-cell lung cancer (NSCLC) [20]. Several trials explored the efficacy of anti-HER2 therapy in metastatic UC. The HER2 tyrosine kinase inhibitors (TKI) (e.g., afatinib, lapatinib, and neratinib) showed an ORR of < 10% and unsatisfactory DCR [11, 21]. Although some case reports suggested durable remission with trastuzumab and chemotherapy in refractory patients, phase II RCTs failed to show the benefit of adding trastuzumab to chemotherapy [22–24]. Dual HER2 blockade of trastuzumab and pertuzumab showed an ORR of only 18% [25]. Therefore, it appears that anti-HER2 antibodies or TKIs have only limited efficacy in metastatic UC. Nevertheless, HER2-based ADC showed promising results. Indeed, trastuzumab deruxtecan, in combination with nivolumab, demonstrated an ORR of 36.7% and PFS of 6.9 months in HER2-positive metastatic UC [26]. In two prospective studies of RC48, the ORR was 51.2–75% [13, 14]. Nevertheless, in the RC48-C005 trial, 72.1% of the patients had received only one line of prior systemic therapy, while more than half of the patients in the RC48-C014 trial were systemic therapy-naïve. In the present real-world study, almost 50% of the patients received at least two lines of prior therapy, and 52.8% had visceral metastasis, supporting the favorable and durable efficacy of RC48 in patients with refractory and resistant metastatic UC after multiple lines of therapy. Notably, among the nine patients who received RC48 as first-line treatment, eight were cisplatin or platinum ineligible and the achieved ORR of 55.6% and PFS time of 6.6 months were promising. Actually, a phase 3 study of RC48 combined with toripalimab for first-line treatment of mUC is undergoing (NCT05302284).

Anticancer therapy for patients with impaired renal function is extremely complicated [27, 28], especially for patients with metastatic UC. The patients with metastatic UC with a performance status ≥ 2, impaired renal function (CrCl 30–59 mL/min), and grade 2 or worse hearing loss were considered cisplatin-intolerant. Carboplatin, EV, and immunotherapy are alternative treatments for cisplatin-ineligible patients [2]. Unfortunately, EV is not available in China. In the present study, only two patients received EV with one participation in the EV 203 clinical trial. In addition, it was reported that > 60% of the patients treated with EV had treatment-related skin reactions, and about 50% had peripheral sensory neuropathy; therefore, the toxicity of EV is an important issue [12]. Immunotherapy, including PD-1 and PD-L1 inhibitors, has a more favorable safety and tolerability profile than chemotherapy (e.g., carboplatin-based regimens), but the ORR is only 20–30% [4]. In the RC48-C005 and RC48-C014 trials, an inclusion criterion was serum creatinine ≤ 1.5 folds the upper normal limit or CrCl ≥ 50 mL/min, as calculated by the Cockcroft Gault equation [13, 14]. Still, many patients suffer from renal insufficiency because of platinum based-chemotherapy, disease progression, or nephrectomy. The treatment of these patients is difficult and under case-by-case exploration. The present study suggested that RC48 was safe in patients with impaired renal function. Five patients with a borderline CrCl of 30 mL/min achieved a durable response and stable renal function. Based on the pharmacokinetics of RC48, the serum concentration is low [29], and the CrCl does not influence the pharmacokinetics and pharmacodynamics of RC48. Thus, it is suggested that the administration of RC48 is safe among patients with moderate or even worse renal impairment. Besides, our study also enrolled several elderly patients over 75 years old, and six patients with performance status of 2; all these patients tolerated RC48 very well, supporting the good tolerance of this drug.

HER2 mutations are detected in 5–14% of patients with metastatic UC [30]. The most common mutation of HER2 is S310F, accounting for 23% [30]. Still, few reports focused on the efficacy of anti-HER2 treatment in patients with HER2-mutated metastatic UC. In the present study, one patient with a HER2 insertion mutation at exon 20 was treated with RC48. The symptoms rapidly improved but gradually aggravated, indicating a difficult case. The proper treatment and the role of HER2-targeted therapy for patients with HER2 mutation are uncertain. The HER-2 overexpression was not completely consistent to HER-2 gene amplification. The concordance between HER2 IHC 3 + and gene amplification by FISH was approximately 70% in UC [31]. One study reported that HER2 gene amplification by NGS or FISH was detected in only 15 patients among 41 patients with HER2 IHC 2 + [32]. The concordance between HER2 IHC and NGS was also not completely consistent in our study. Further exploration in our future study was warranted. Combining ADC and immunotherapy seems promising, but more evidence for the synergistic effect needs exploration [33]. The combination of EV and pembrolizumab showed a promising PFS of 12.3 months in first-line cisplatin-ineligible patients [34], while similar PFS was observed between SG monotherapy or combined with pembrolizumab in the TROPHY-U-01 trial [9, 35]. The synergistic effect of HER2 ADC and PD-1 inhibitor varied among studies. The combination of trastuzumab deruxtecan (T-DXd) with nivolumab presented an ORR of 36.7% [26], while the combination of RC48 and toripalimab in the RC48-C014 trial showed an ORR of 75.0% [14]. Nevertheless, the comparison between RC48-ADC alone and the combination of immunotherapy is lacking. In the present study, the addition of immunotherapy might present a longer PFS compared with RC48-ADC alone, but the differences did not reach statistical significance. Therefore, a future study might focus on the proper combination strategy, potential benefits, and which patient population might benefit the most.

The limitations of this study lie in its retrospective nature and the heterogeneity in baseline characteristics and treatment factors, which might lead to potential bias. Furthermore, only eight out of the 36 patients underwent NGS, and more genomic information is needed in the future. The main strength of the present study was that it analyzed the efficacy and safety of RC48 alone or in combination with immunotherapy in advanced UC, especially in patients with impaired renal function. Therefore, more prospective clinical trials for RC48 in a larger sample size are warranted for confirmation.

In conclusion, RC48 alone or combined with immunotherapy showed excellent antitumor activity in patients with locally advanced or metastatic UC. RC48 might be safe even in patients with moderate or severe renal failure. A phase III RCT is warranted to compare RC48-ADC alone and in combination with immunotherapy.

### Supplementary Information

Below is the link to the electronic supplementary material.

Figure S1. Progression-free survival (PFS) of the patients receiving RC48 as first line, second and later line therapy (TIF 18800 KB)

Table S1. List of patients received first-line RC48 and not received platinum-based therapy (DOCX 15 KB)

## Data Availability

The datasets generated during the current study are available from the corresponding author upon reasonable request.

## References

[1] Patel VG, Oh WK, Galsky MD (2020). Treatment of muscle‐invasive and advanced bladder cancer in 2020. CA: A Cancer J Clin.

[2] NCCN Clinical Practice Guidelines in Oncology (NCCN Guidelines) (2022) Bladder cancer. Version 2.2022. National Comprehensive Cancer Network, Fort Washington

[3] De Santis M, Bellmunt J, Graham Mead J, Kerst M, Leahy M, Maroto P (2012). Randomized phase II/III trial assessing gemcitabine/carboplatin and methotrexate/carboplatin/vinblastine in patients with advanced urothelial cancer who are unfit for cisplatin-based chemotherapy: EORTC study 30986. J Clin Oncol.

[4] Vuky J, Balar AV, Castellano D, O’Donnell PH, Grivas P, Bellmunt J (2020). Long-term outcomes in KEYNOTE-052: Phase II Study investigating first-line pembrolizumab in cisplatin-ineligible patients with locally advanced or metastatic urothelial cancer. J Clin Oncol.

[5] Chang SS, Bochner BH, Chou R, Dreicer R, Kamat AM, Lerner SP (2017). Treatment of non-metastatic muscle-invasive bladder cancer: AUA/ASCO/ASTRO/SUO guideline. J Urol.

[6] Bellmunt J, de Wit R, Vaughn D, Fradet Y, Lee J, Fong L (2017). Pembrolizumab as second-line therapy for advanced urothelial carcinoma. N Engl J Med.

[7] Sharma P, Retz M, Siefker-Radtke A, Baron A, Necchi A, Bedke J (2017). Nivolumab in metastatic urothelial carcinoma after platinum therapy (CheckMate 275): a multicentre, single-arm, phase 2 trial. Lancet Oncol.

[8] Powles T, Rosenberg J, Sonpavde G, Loriot Y, Durán I, Lee J (2021). Enfortumab vedotin in previously treated advanced urothelial carcinoma. N Engl J Med.

[9] Tagawa ST, Balar AV, Petrylak DP, Kalebasty AR, Loriot Y, Fléchon A (2021). TROPHY-U-01: a Phase II Open-Label Study of Sacituzumab govitecan in patients with metastatic urothelial carcinoma progressing after platinum-based chemotherapy and checkpoint inhibitors. J Clin Oncol.

[10] Yorozu T, Sato S, Kimura T, Iwatani K, Onuma H, Yanagisawa T (2020). HER2 status in molecular subtypes of urothelial carcinoma of the renal pelvis and ureter. Clin Genitourin Cancer.

[11] Patelli G, Zeppellini A, Spina F, Righetti E, Stabile S, Amatu A (2022). The evolving panorama of HER2-targeted treatments in metastatic urothelial cancer: a systematic review and future perspectives. Cancer Treat Rev.

[12] Yu EY, Petrylak DP, O'Donnell PH, Lee J-L, van der Heijden MS, Loriot Y (2021). Enfortumab vedotin after PD-1 or PD-L1 inhibitors in cisplatin-ineligible patients with advanced urothelial carcinoma (EV‑201): a multicentre, single-arm, phase 2 trial. Lancet Oncol.

[13] Sheng X, Yan X, Wang L, Shi Y, Yao X, Luo H (2021). Open-label, multicenter, Phase II Study of RC48-ADC, a HER2-targeting antibody–drug conjugate patients with locally advanced or metastatic urothelial carcinoma. Clin Cancer Res.

[14] Zhou L, Huayan X, Li S, Yan X, Li J, Xiaowen W (2022). Study RC48-C014: preliminary results of RC48-ADC combined with toripalimab in patients with locally advanced or metastatic urothelial carcinoma. J Clin Oncol.

[15] Kim H-S, Suehyun Lee J, Kim H (2018). Real-world evidence versus randomized controlled trial: clinical research based on electronic medical records. J Korean Med Sci.

[16] Eichler HG, Pignatti F, Schwarzer-Daum B, Hidalgo-Simon A, Eichler I, Arlett P (2021). Randomized controlled trials versus real world evidence: neither magic nor myth. Clin Pharmacol Ther.

[17] Zhang H, Moisini I, Ajabnoor RM, Turner BM, Hicks DG (2020). Applying the new guidelines of HER2 testing in breast cancer. Curr Oncol Rep.

[18] Chen D, Ye Y, Guo S, Yao K (2021). Progress in the research and targeted therapy of ErbB/HER receptors in urothelial bladder cancer. Front Mol Biosci.

[19] Pellino A, Riello E, Nappo F, Brignola S, Murgioni S, Djaballah SA (2019). Targeted therapies in metastatic gastric cancer: current knowledge and future perspectives. World J Gastroenterol.

[20] Zhao J, Xia Y (2020). Targeting HER2 alterations in non-small-cell lung cancer: a comprehensive review. JCO Precis Oncol.

[21] Choudhury N, Campanile A, Antic T, Yap K, Fitzpatrick C, Wade J (2016). Afatinib activity in platinum-refractory metastatic urothelial carcinoma in patients with ERBB alterations. J Clin Oncol.

[22] Wezel F, Erben P, Gaiser T, Budjan J, von Hardenberg J, Michel MS (2018). Complete and durable remission of human epidermal growth factor receptor 2-positive metastatic urothelial carcinoma following third-line treatment with trastuzumab and gemcitabine. Urologia Int.

[23] Jiang Q, Xie M-X, Zhang X-C (2020). Complete response to trastuzumab and chemotherapy in recurrent urothelial bladder carcinoma with HER2 gene amplification: a case report. World J Clin Cases.

[24] Oudard S, Culine S, Vano Y, Goldwasser F, Théodore C, Nguyen T (2015). Multicentre randomised phase II trial of gemcitabine + platinum, with or without trastuzumab, in advanced or metastatic urothelial carcinoma overexpressing Her2. Eur J Cancer.

[25] Takahashi K, Ishibashi E, Kubo T, Harada Y, Hayashi H, Kano M (2020). A phase 2 basket trial of combination therapy with trastuzumab and pertuzumab in patients with solid cancers harboring human epidermal growth factor receptor 2 amplification (JUPITER trial). Medicine.

[26] Galsky MD, Del Conte G, Foti S, Yu EY, Machiels J-PH, Doger B (2022). Primary analysis from DS8201-A-U105: a phase 1b, two-part, open-label study of trastuzumab deruxtecan (T-DXd) with nivolumab (nivo) in patients (pts) with HER2-expressing urothelial carcinoma (UC). J Clin Oncol.

[27] Shahinian VB, Bahl A, Niepel D, Lorusso V (2017). Considering renal risk while managing cancer. Cancer Manag Res.

[28] Hendrayana T, Wilmer A, Kurth V, Schmidt-Wolf IG, Jaehde U (2017). Anticancer dose adjustment for patients with renal and hepatic dysfunction: from scientific evidence to clinical application. Sci Pharm.

[29] Shi F, Liu Y, Zhou X, Shen P, Xue R, Zhang M (2022). Disitamab vedotin: a novel antibody–drug conjugates for cancer therapy. Drug Deliv.

[30] Madison RW, Gupta SV, Elamin YY, Lin DI, Pal SK, Necchi A (2020). Urothelial cancer harbours EGFR and HER2 amplifications and exon 20 insertions: EGFR/ERBB2 exon 20 insertions. BJU Int.

[31] Scherrer E, Kang A, Bloudek LM, Koshkin VS (2022). HER2 expression in urothelial carcinoma, a systematic literature review. Front Oncol.

[32] Li S, Xiaowen W, Yan X, Zhou L, Huayan X, Li J (2022). Prognostic value of HER2 expression levels for upper tract urothelial carcinoma. J Clin Oncol.

[33] Nicolò E, Giugliano F, Ascione L, Tarantino P, Corti C, Tolaney S (2022). Combining antibody–drug conjugates with immunotherapy in solid tumors: current landscape and future perspectives. Cancer Treat Rev.

[34] Friedlander TW, Milowsky MI, Bilen MA, Srinivas S, McKay RR, Flaig TW (2021). Study EV-103: update on durability results and long term outcome of enfortumab vedotin + pembrolizumab in first line locally advanced or metastatic urothelial carcinoma (la/mUC). J Clin Oncol.

[35] Grivas P, Pouessel D, Park CH, Barthélémy P, Bupathi M, Petrylak DP (2022). TROPHY-U-01 cohort 3: Sacituzumab govitecan (SG) in combination with pembrolizumab (Pembro) in patients (pts) with metastatic urothelial cancer (mUC) who progressed after platinum (PLT)-based regimens. J Clin Oncol.

