# Supplementary figures and images for: HER2-targeting antibody–drug conjugate RC48 alone or in combination with immunotherapy for locally advanced or metastatic urothelial carcinoma: a multicenter, real-world study
Source: Cancer Immunol Immunother. 2023 Mar 10;72(7):2309–18. doi: 10.1007/s00262-023-03419-1 (PMC10264489; doi:10.1007/s00262-023-03419-1)

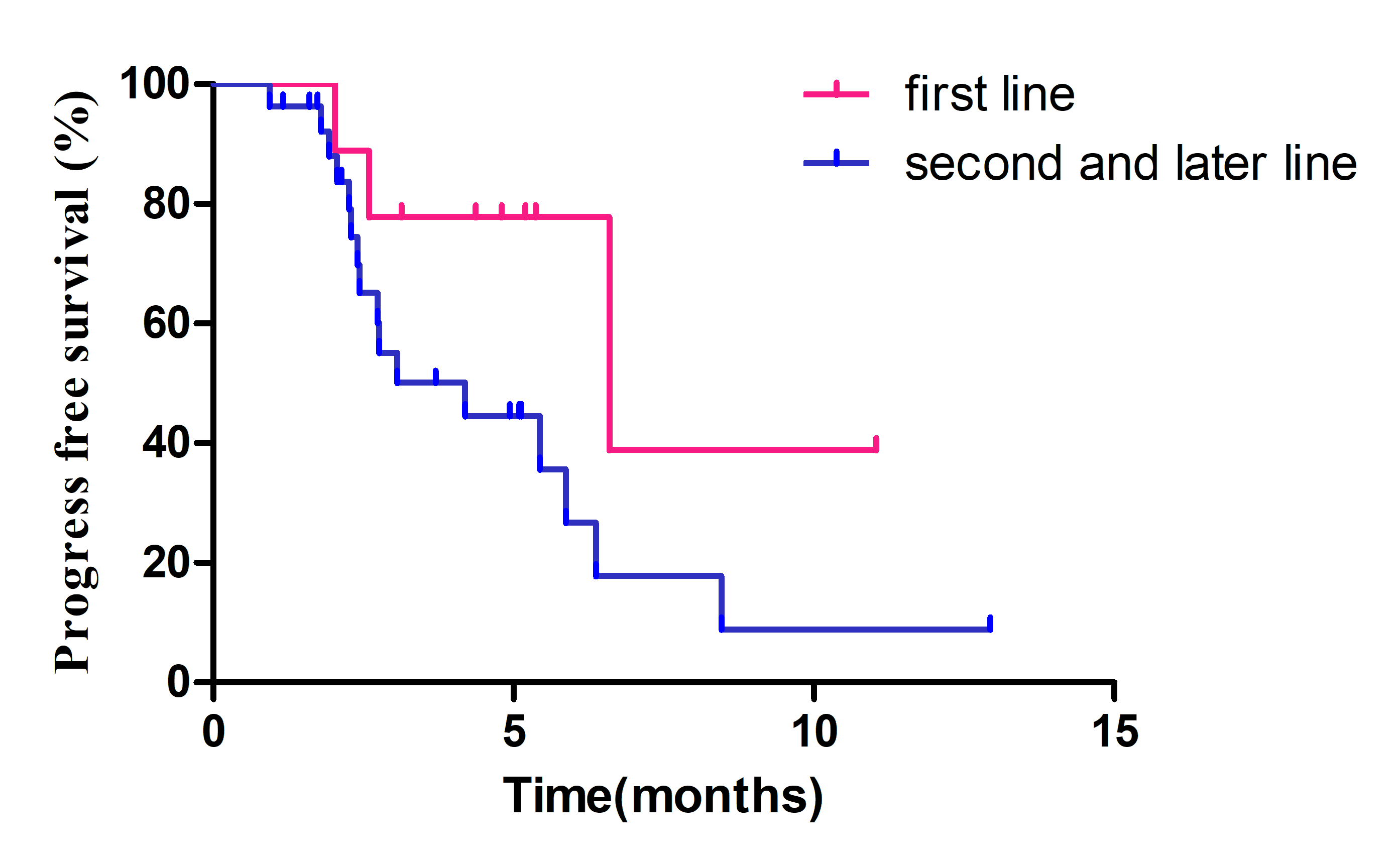

Supplement: Supplementary file 1 — Figure S1. Progression-free survival (PFS) of the patients receiving RC48 as first line, second and later line therapy (TIF 18800 KB) [file 262_2023_3419_MOESM1_ESM.tif]
