# Supplementary material for: HER2-targeting antibody–drug conjugate RC48 alone or in combination with immunotherapy for locally advanced or metastatic urothelial carcinoma: a multicenter, real-world study
Source: Cancer Immunol Immunother. 2023 Mar 10;72(7):2309–18. doi: 10.1007/s00262-023-03419-1 (PMC10264489; doi:10.1007/s00262-023-03419-1)
Supplement: Supplementary file 2 — Table S1. List of patients received first-line RC48 and not received platinum-based therapy (DOCX 15 KB) [file 262_2023_3419_MOESM2_ESM.docx]

Table S1 List of patients received first-line RC48 and not received platinum-based therapy

| Patient No. | **Reasons for first-line RC48** |
| --- | --- |
| #1 | She had a history of renal transplantation, and ineligible for immunotherapy. Her poor renal function resulted in ineligibility for any platinum-based chemotherapy. |
| #6 | She was ineligible for cisplatin-based chemotherapy due to poor renal function, and had PD-L1 IC 10% and TC 2%, HER2 2+ disease |
| #18 | Ineligible for cisplatin-based chemotherapy due to poor renal function, and had HER2 2+ disease |
| #20 | 70 years old female, refused to platinum-based chemotherapy, and had HER2 IHC 2+ disease. |
| #21 | Ineligible for cisplatin -based chemotherapy due to poor renal function, with history of creatinine elevation after bladder perfusion of adriamycin and had HER2 1+ disease. He strongly refused systemic chemotherapy. |
| #22 | Ineligible for cisplatin-based chemotherapy due to poor renal function, and had PD-L1 negative, and HER2 2+ disease. |
| #24 | Ineligible for any platinum-based chemotherapy due to age of 81 year-old, poor renal function, and multiple comorbidities. He had HER2 2+ disease. |
| #29 | Ineligible for cisplatin-based chemotherapy due to poor performance status of 2, and had HER2 3+ disease |
| #36 | Ineligible for any platinum-based chemotherapy due to old age (87y), multiple comorbidities, with HER2 3+ disease. |
| Patient No. | **Reasons for not received platinum-based chemotherapy** |
| #7 | Ineligible for cisplatin-based chemotherapy due to poor renal function. He enrolled in a clinical trial and received gemcitabine combined with nab-paclitaxel as first-line therapy. He had PD-L1 negative, HER2 2+ disease. |
| #17 | Ineligible for cisplatin-based chemotherapy due to poor renal function, and had HER2 2+ disease. He relapsed after adjuvant therapy of PD-1 antibody. |
| #27 | 72y-old male relapsed after adjuvant therapy. He was ineligible for cisplatin-based chemotherapy. |
| #35 | 78y-old man ineligible for any platinum-based chemotherapy due to poor renal function, and he progressed after first-line therapy of PD-1 immunotherapy. |
